# Tears or Fears? Comparing Gender Stereotypes about Movie Preferences to Actual Preferences

**DOI:** 10.3389/fpsyg.2017.00428

**Published:** 2017-03-24

**Authors:** Peter Wühr, Benjamin P. Lange, Sascha Schwarz

**Affiliations:** ^1^Department of Psychology, TU Dortmund UniversityDortmund, Germany; ^2^Department of Media Psychology, Julius Maximilian University of WuerzburgWuerzburg, Germany; ^3^Department of Psychology, University of WuppertalWuppertal, Germany

**Keywords:** media psychology, gender, movie genre, stereotype, stereotype accuracy

## Abstract

This study investigated the accuracy of gender-specific stereotypes about movie-genre preferences for 17 genres. In Study 1, female and male participants rated the extent to which 17 movie genres are preferred by women or men. In Study 2, another sample of female and male participants rated their own preference for each genre. There were three notable results. First, Study 1 revealed the existence of gender stereotypes for the majority of genres (i.e., for 15 of 17 genres). Second, Study 2 revealed the existence of actual gender differences in preferences for the majority of genres (i.e., for 11 of 17 genres). Third, in order to assess the accuracy of gender stereotypes on movie preferences, we compared the results of both studies and found that the majority of gender stereotypes were accurate in direction, but inaccurate in size. In particular, the stereotypes overestimated actual gender differences for the majority of movie genres (i.e., 10 of 17). Practical and theoretical implications of these findings are discussed.

## Introduction

Many people have gender stereotypes. Gender stereotypes are beliefs concerning the abilities, attitudes, preferences and behaviors of a ‘typical’ man or a ‘typical’ woman (Mealey, 2000). For example, many people believe that women have better verbal abilities than men, whereas men are assumed to have better mathematical skills than women. Whereas research has partly confirmed the former assumption (e.g., for verbal fluency, see Hyde and Linn, 1988), it has disconfirmed the latter (cf. Hyde, 2016, for a review). Because stereotypes may, at least partly, reflect real differences between groups, stereotypes are not necessarily inaccurate (see Hilton and von Hippel, 1996; Brown, 2010, for reviews on stereotypes in general). Hence, “the accuracy of stereotypes is an empirical question, not an ideological one” (Jussim et al., 2009, p. 199). Previous research has already addressed the accuracy of gender stereotypes concerning attitudes (e.g., Diekman et al., 2002) or cognitive abilities (e.g., Halpern et al., 2011; Hyde, 2016), but there is almost no research about the accuracy of gender stereotypes concerning media preferences. The present study thus compared presumed and actual differences in film preferences of men and women.

### Gender and Movie Preferences

Everyday observations suggest the existence of strong and well-known stereotypes about gender differences in movie preferences, which can be summarized as follows. Women are supposed to like romantic and melodramatic movies, which are snidely called “chick flicks” or “tearjerkers”, as well as comedy, but to dislike action and horror movies. In contrast, men are supposed to like action and horror movies, but to dislike romantic and melodramatic movies. Empirical research on gender differences in actual movie preferences has confirmed, at least to some degree, these popular stereotypes (see Oliver, 2000; Greenwood and Lippmann, 2010, for reviews). The assumption that women prefer romantic and melodramatic movies as well as comedy, for instance, has received some empirical support (e.g., Oliver et al., 1998; Harris et al., 2000). The same is evident for the larger male than female preference for action and horror movies (e.g., Sparks, 1991; Krcmar and Kean, 2005).

Greenwood (2010), for example, investigated the impact of gender and mood state (happy vs. sad) on choosing between films from different genres. First, participants had to write about a happy or sad life event in order to induce the corresponding mood in these participants. Then, participants were asked to indicate which type of film they wished to see right now by using closed and open questions. The questionnaire required a choice between some happy film genres (e.g., romantic comedy, dark comedy), some sad film genres (e.g., social drama, romantic drama), action-adventure movies, and suspenseful movies (i.e., thriller). Results showed that, independent from induced mood, female participants preferred romantic genres (i.e., romantic comedy, romantic drama), whereas male participants preferred action-adventure movies and suspenseful films.

Further results showed that men, in stark contrast to women, have a preference for programs with violent or sexual content. For example, Blanchard et al. (1986) presented their participants violent material from different sources or genres (i.e., from a spy movie, a war movie, a western movie, and a cartoon). When interviewed afterwards, male participants reported more pleasure from viewing the violent films than the female participants (see also Koukounas and McCabe, 2001). A stronger male than female preference was also found for horror movies that typically contain frightening and violent scenes (e.g., Cantor and Reilly, 1982; Zillmann et al., 1986; Oliver, 1993).

Finally, strong gender differences have also been observed in the use and reception of pornographic material. A couple of studies revealed that men consume pornographic films and materials more frequently than women (e.g., Bryant and Brown, 1989; Hald, 2006; see Peter and Valkenburg, 2016, for reviews). Moreover, men repeatedly reported stronger affective and physiological responses to pornographic films than did women (e.g., Janssen et al., 2003). However, the size of the difference between male and female responses to pornographic material has been found to vary with some characteristics of the films (e.g., Murnen and Stockton, 1997; Janssen et al., 2003; Hald and Štulhofer, 2015).

To summarize, previous studies on film and movie genre preferences have already discovered some reliable gender differences, as described above. However, most of these studies involved relatively small numbers of movie genres (i.e., 2–4; e.g., Sparks, 1991; Oliver et al., 1998), and have focused on a relatively small subset of genres, including action, drama, horror, sports, and pornography. Moreover, in most studies, participants judged parts of individual films or verbal descriptions of fictive films, and the representativeness of these materials constrains any attempts to generalize the results to the genre in question. For these reasons, we do not yet know much about some movie genres (e.g., fantasy films, history films, mystery films, science-fiction films, crime movies, western films and, as a typical German genre, ‘heimat’ films).^1^ And we do not yet know much about the relative popularity of movie genres within and across both sexes, either. Finally, studies typically addressed the actual preferences of men and women for movie genres, whereas gender stereotypes in movie preferences have neither been studied nor have stereotypes been compared to the actual movie preferences of men and women.

### Possible Sources of Gender Stereotypes

Although the data presented here are not suitable for answering the question of where gender stereotypes and gender differences with respect to media preferences come from, it might be worthwhile to summarize the most prominent accounts of gender differences in movie preferences. These accounts can be classified in four groups: theories referring to media content, biological accounts, evolutionary accounts, and accounts in terms of gender socialization (e.g., Oliver, 2000; Hust and Brown, 2008).

A first group of accounts refers to differences in the content of films from different genres, such as the main topic of the film or the protagonists’ gender. Regarding film topics, it was suggested that women prefer movies or genres centering on social relationships, whereas men prefer movies or genres centering on aggressive conflicts (e.g., Oliver et al., 2000). Regarding the gender of film protagonists, it was suggested that observers especially enjoy films if they can identify with a major protagonist, and observers prefer to identify with same-sex protagonists (e.g., Hoffner and Cantor, 1991; Hoffner, 1996). In fact, content analyses showed that male protagonists dominate in film genres that are preferred by men (e.g., action films, ‘male’ sports), whereas female protagonists are more likely to be found in film genres that are preferred by women (e.g., daily soaps, melodramatic movies).

Biological accounts refer to biological differences between men and women (e.g., Malamuth, 1996; Goldstein, 1998). For example, the male hormone testosterone is related to sex drive and dominant behavior in men (e.g., Mazur and Booth, 1998). This relationship could provide an account for the stronger interest of men, compared to women, in movies featuring competition and sex. The female hormones oxytocin and vasopressin, however, are supposed to be related to pair bonding and feelings of love in humans (e.g., Fisher, 1998; Carter, 2007). From a neurobiological perspective this difference could be responsible for women’s greater interest in romantic movies. It should be noted though that biological accounts are controversially discussed. For example, researchers from the field of “gendered neurocultures” argue that biological accounts should critically be reflected in terms of gendered ascriptions that are linked to biological processes (e.g., Fillod, 2014; Matusall, 2014).

Evolutionary accounts describe how human evolution may have shaped gender differences in media preferences. For example, from parental investment theory (Trivers, 1972), it can be predicted that “women prefer media contents dealing with issues of mate choice, partner loyalty, and the loss of a partner … [and] men are more likely to select media contents dealing with kin protection, rivalry, status, power, and the acquisition and maintenance of resources” (Hennighausen and Schwab, 2015, p. 144). In a sample of movie visitors, Schwab (2010) replicated the finding that women prefer melodramatic and romantic movies, whereas men prefer action movies. Interestingly, and in some contrast to previous findings, Schwab observed no significant gender differences for other genres (i.e., comedy, horror, and thriller).

A fourth group of accounts refers to differences in the socialization of men and women and, as a result, to differences in learned gender roles. Two important theories of gender socialization are social-learning theory and gender-schema theory. According to social learning theory, children learn the gender role matching their biological sex by operant conditioning and/or by latent learning (e.g., Mischel, 1966). Hence, the agents of socialization (e.g., parents, peers, and teachers) are assumed to reward children for consuming gender-role congruent media content, and to punish (or, at least, refrain from rewarding) children for consuming gender-role incongruent media content. In addition, children may also learn gendered media preferences by observing and imitating same-sex role models. Whereas social-learning theory focuses on the acquisition of gendered behavior, gender-schema theory focuses on the cognitive representation of the gender role. A gender schema is a cognitive structure representing knowledge about the (typical) features of a gender (e.g., Bem, 1981). During socialization, children acquire gender schemas and form associations between the contents of their self-concept and the contents of the gender schema matching their biological sex. Importantly, the gender schema not only represents knowledge about gender, but it also biases the processing of information towards the own gender. Hence, on this account, boys and girls develop different media preferences because (different) gender schemas direct their attention towards gender-congruent media contents.

Presumably, not a single account is able to explain gender differences in media preferences in their entirety. More likely, a combination of different accounts provides the most valid explanation. For example, it might be that men and women are born with some innate biological cognitive mechanisms that are the result of human evolution. Those mechanisms might then be further shaped by environmental factors and thus, finally, cause the observed preferences, for instance for a specific movie content.

### Accuracy of Gender Stereotypes

Only recently, researchers have begun to empirically assess the accuracy of social stereotypes, and to compare stereotypes about groups and group differences to measurements of group attributes and group differences (see Ryan, 2002; Jussim et al., 2009, for reviews). The majority of these studies revealed that people are able to provide accurate judgments for many attributes of men and women (Hall and Carter, 1999; Jussim et al., 2009). For example, Swim (1994) compared stereotypes about gender differences for 17 characteristics (e.g., aggression, cognitive abilities, non-verbal behaviors) to measures of actual gender differences as derived from meta-analytic studies. She observed accurate judgments of gender differences for the majority of characteristics under investigation. The only overestimations of gender differences occurred for aggression and verbal abilities. Similarly, Diekman et al. (2002) reported that, on average, male and female participants quite accurately predicted the political and social attitudes of men and women.

The evidence on the accuracy of people’s beliefs about gender differences in cognitive abilities appears somewhat mixed, however. Beyer (1999), for example, found that university students misperceived the direction of the gender difference in performance (i.e., the grade point average, GPA) for a set of ‘masculine’ majors^2^: Participants believed that male students perform better in these majors, although female students actually achieved higher grades. As already stated, Swim (1994) reported that participants quite accurately judged (existing) gender differences in math performance, but overestimated gender differences in tests on verbal abilities. Finally, Halpern et al. (2011) found that participants were generally accurate about the direction of gender differences in the cognitive domain, but underestimated the actual size of the differences. In their recent review on the accuracy of gender stereotypes, Jussim et al. (2009, p. 211) conclude that, “at least a plurality of judgments was accurate” and that “there was no support for the hypothesis that stereotypes generally lead people to exaggerate real differences.”

Determining the accuracy of stereotypes in general and of gender stereotypes in particular is important for at least three reasons. First, assessing and analyzing the accuracy of stereotypes may provide important clues on the origins and development of stereotypes. For example, Hall and Carter (1999) identified several abilities and traits (e.g., interpersonal sensitivity) that were correlated with the accuracy of gender stereotypes. Second, the evidence demonstrating that a stereotype is wrong could (and should) be used to correct this stereotype and to improve the relationship between social groups (e.g., Beyer, 1999). Third, comparing presumed preferences (i.e., stereotypes) to the actual preferences of social groups may provide helpful information for some industries, including the movie industry in case movie preferences are concerned. In particular, knowledge about discrepancies between presumed and actual gender differences in movie preferences may be helpful for addressing a broader audience. If, for example, the actual gender gap in preference for a particular movie genre is larger than presumed, one might reduce this difference by producing more gender-neutral movies of this genre. Also, movie producers might benefit from knowing the actual size of the gender gap by focusing more on producing gender-typical movies.

### The Present Study

The present investigation had four aims. The first aim was to investigate—for the first time (at least to our knowledge)—the stereotypes of men and women about the movie preferences of men and women (Study 1). The second aim was to investigate gender differences in stereotypes about the movie preferences of men and women (Study 1). In other words, we wanted to investigate whether men and women have similar or different stereotypes about gender-specific movie preferences. The third aim of our study was to investigate the actual preferences of young adults for our set of movie genres (Study 2). Hence, we addressed more genres than most of the previous studies, and included genres that have not been investigated (often) before (e.g., ‘heimat’, history, mystery, crime, thriller, and western). Finally, the fourth aim of our study was assessing the accuracy of gender stereotypes concerning movie preferences by comparing the results of Study 1 to those of Study 2.

We assessed and compared presumed gender differences in movie preferences to actual gender differences in movie preferences for 17 movie genres. The term “genre” refers to a category of motion pictures that are similar with regard to particular features, including topic (e.g., adventure, war), geographical or historical background (e.g., Western, science fiction), stylistic issues (e.g., animation) and the targeted audience (e.g., children vs. adults; cf. Grant, 2003). For our investigation, we chose 17 well-known movie genres^3^: (1) action movie, (2) adventure movie, (3) animation movie, (4) comedy movie, (5) crime movie, (6) drama, (7) erotic movie, (8) fantasy movie, (9) ‘heimat’ film, (10) historic movie, (11) horror movie, (12) mystery movie, (13) romance, (14) science fiction movie, (15) thriller movie, (16) war movie, (17) Western movie. Only the genre names were used in both studies. We did not present particular examples for each genre because we wanted to investigate how participants think about movie genres, and not how they think about particular movies, and we had several additional reasons for doing that. First, we were sure that our participants would recognize each of the 17 movie genres used in our study without providing examples. Second, the representativeness of a particular movie for a genre is always debatable. Third, example movies might be familiar to some participants, but not to all. Hence, providing no examples created equal conditions for all participants. Finally, we deliberately chose genres of movies that are typically presented in the cinema (and on TV). Hence, we excluded common TV formats like daily soaps, documentaries, sports, talk shows, sitcoms and so on.

## Study 1: Stereotypes About Movie Preferences Of Men And Women

In Study 1, we investigated the stereotypes of young adults about movie genre preferences of men and women in order to address two research questions. First, we asked which genres are generally associated with men and which genres are generally associated with women. For some genres, we expected to observe the popular gender stereotype. In particular, we expected that a majority of participants would ascribe romantic movies and drama to women. Similarly, we expected that a majority of participants would ascribe more action-packed and violent genres (i.e., action, adventure, horror, thriller, and war movies) to men. We had no predictions regarding the remaining genres. Our second research question asked whether male and female participants would express similar or different views (i.e., stereotypes) about the movie preferences of men and women.

### Materials and Methods

#### Ethics, Consent, and Permissions

The research reported in our manuscript meets the ethical guidelines of the German Society of Psychology (Deutsche Gesellschaft für Psychologie, DGPs) and is consistent with the principles of research ethics as published by the American Psychological Association (APA). Data collection was completely anonymous. That is, except for age and gender, we did not record personal information from the participants. All participants in the two studies reported here gave informed consent before filling out the questionnaires on movie preferences. The research reported here had not been approved by a local ethics committee because the ethical guidelines of the German Society of Psychology do not require ethical approval of basic psychological studies involving simple behavioral data.

#### Participants and Interviewer

One hundred and fifty volunteers participated in Study 1. The sample consisted of 75 women (mean age = 23.1 years; *SD* = 4.0) and 75 men (mean age = 24.1 years, *SD* = 3.8). The large majority of participants were students (undergraduates) at a public university in Germany. The interviewers were 15 students (12 female, 3 male), and each interviewed 5 men and 5 women.

#### Materials

We developed a questionnaire with 24 items^4^. Items 1–4 asked for some demographic characteristics of our participants (gender, age, main profession or major study course, minor study course). Then, items 5–21 asked the participant to indicate for each of 17 movie genres the degree to which the respective genre is, in her or his opinion, preferred by men or women. In particular, each item asked: “What do you think: Are (adventure) films preferred by men or by women?” Participants had to indicate their answer on an 11-point rating scale. The ticks were numbered from 5 to 0 and to 5 again. The central tick (0) was always marked as “equally preferred by men and women”. For half of the questionnaires (i.e., participants) the leftmost tick was marked as “exclusively preferred by women”, whereas the rightmost tick was marked as “exclusively preferred by men”. For the other half of questionnaires (i.e., participants) the leftmost tick was marked as “exclusively preferred by men”, whereas the rightmost tick was marked as “exclusively preferred by women”. The items 5–21 probed for the following movie genres (the German terms are given in brackets): adventure [Abenteuerfilm], action [Action-Film], animation [Animationsfilm], comedy [Filmkomödie], crime [Kriminalfilm], drama [Filmdrama], erotic [Erotikfilm], fantasy [Fantasy-Film], ‘heimat’ [Heimatfilm], history [Historienfilm], horror [Horrorfilm], romance [Liebesfilm], mystery [Mystery-Film], science fiction [Science-Fiction-Film], thriller [Thriller], war [Kriegsfilm], and Western [Westernfilm]. The items 22–24 asked our participants for some quantitative aspects of TV and film consumption. Item 22 asked how many hours per day, on average, the participant watched TV. Item 23 asked how many films, on average, the participant consumed per week. Finally, item 24 asked how often, on average, the participant went to the cinema per month.

We constructed four versions of our questionnaire in order to minimize (a) the effects of marker positions and (b) the effects of item order. As already said above, in half of the questionnaires the left pole of the rating scales referred to women, whereas the right pole referred to men, and the reverse was true for the other half of questionnaires. This manipulation was crossed with two different orders of movie genres. For one half of the questionnaires, items 5–21 were jumbled to produce a random order of genres. This (random) order was reversed for the other half of the questionnaires. We made sure that similar numbers of men and women filled out each version of the questionnaire.

#### Procedure

People were asked to participate in a study on the film preferences of men and women. Participants filled out the questionnaire on themselves, in order to minimize interviewer effects. If necessary, the interviewer provided a pen and a writing pad. Data were always collected in dyads of one interviewer and one participant. Participants took either part for free or received course credits.

### Results

#### Stereotypes about Film Preferences of Men and Women

**FIGURE 1:**
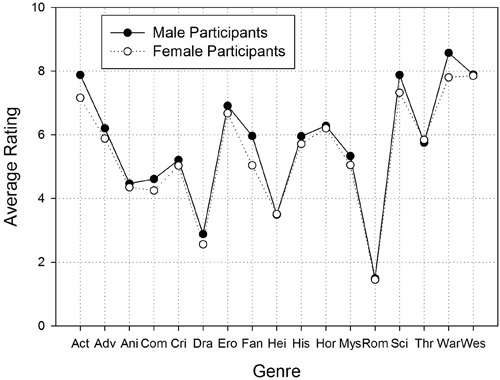
**Results of Study 1: Average ratings from 75 male and 75 female participants, who indicated the degree to which 17 movie genres are preferred by woman or men on a rating scale with 11 ticks ranging from 0 (exclusively preferred by women) to 10 (exclusively preferred by men).** Short-hands on the *x*-axis mean: Act = Action, Adv = Adventure, Ani = Animation, Com = Comedy, Cri = Crime, Dra = Drama, Ero = Erotic, Fan = Fantasy, Hei = Heimat, His = History, Hor = Horror, Mys = Mystery, Rom = Romance, Sci = Science-Fiction, Thr = Thriller, Wes = Western

We first transformed the ratings into numbers ranging from 0 (exclusively preferred by women) to 10 (exclusively preferred by men). The arithmetic means of the ratings are depicted in **Figure 1**. In the next step, we submitted the means to a four-way Analysis of Variance (ANOVA) with the within-subjects factor *Genre*, and the between-subject factors *Gender of Participant*, *Gender of Interviewer*, and *Item Order*^5^. In this analysis, *Gender of Interviewer* and *Item Order* had no significant effects (all *F*s < 1.70, all *p*s > 0.05) and, therefore, we dropped these variables from further analyses. A two-factorial ANOVA revealed significant main effects for *Gender of Participant*, *F*(1,148) = 16.13, *MSE* = 3.55, *p* < 0.001, $\eta_{P}^{2}$ = 0.10, and for *Genre*, *F*(12,1756) = 216.14, *MSE* = 3.19, *p* < 0.001, $\eta_{P}^{2}$ = 0.59. The two-way interaction was not significant, *F*(12,1756) = 1.33, *MSE* = 3.19, *p* = 0.19, $\eta_{P}^{2}$ = 0.05.

The main effect of *Gender of Participant* resulted from the fact that judgments were biased toward the own gender. That is, the women’s judgments for most genres were biased towards the “female” pole, whereas the men’s judgments for most genres were biased towards the “male” pole. In order to clarify the main effect of *Genre*, we compared the mean judgment for each genre to the neutral value of 5 (i.e., the 0 in the questionnaire). Because multiple tests increase the risk of making a *type-1* error, we used Bonferroni’s method to reduce the significance criterion α from 0.05 to 0.003. A significant positive deviation from the neutral value of 5 indicates a ‘male’ genre. Ten genres were viewed as male genres (the results of the individual tests are presented in **Table 1**): action, adventure, erotic, fantasy, history, horror, science fiction, thriller, war, and Western. A significant negative deviation from the neutral value of 5 indicates a ‘female’ genre. Five genres were viewed as female genres: animation, comedy, drama, ‘heimat’ films, and romantic movies. The remaining two genres (i.e., crime and mystery) were perceived as gender neutral.

**Table 1:** Results of *t*-tests involving the mean preference ratings for 17 movie genres.

	(a) Study 1: Gender stereotype				(b) Study 2: Genre popularity				(c) Study 2: Gender preference			
Genre	*t*	*df*	*p*	*g*	*t*	*df*	*p*	*g*	*t*	*df*	*p*	*g*
Action	22.70	149	<0.003	1.85	6.64	159	<0.003	0.52	5.79	152	<0.003	0.91
Adventure	8.54	149	<0.003	0.70	6.75	159	<0.003	0.53	4.76	153	<0.003	0.76
Animation	-4.47	149	<0.003	-0.37	2.84	159	0.005	0.23	1.51	158	0.13	0.24
Comedy	-4.77	149	<0.003	-0.39	14.05	159	<0.003	1.11	1.06	158	0.29	0.17
Crime	1.14	149	0.25	0.09	7.14	159	<0.003	0.57	-1.29	158	0.20	-0.20
Drama	-20.36	149	<0.003	-1.66	2.20	159	0.03	0.17	-3.18	158	<0.003	-0.50
Erotic	10.39	149	<0.003	0.85	-6.79	159	<0.003	-0.54	4.98	148	<0.003	0.78
Fantasy	3.29	149	<0.003	0.27	1.72	159	0.09	0.14	3.35	158	<0.003	0.53
‘Heimat’	-11.16	149	<0.003	-0.91	-13.01	159	<0.003	-1.03	-0.83	158	0.41	-0.13
History	5.49	149	<0.003	0.45	0.12	159	0.91	0.01	-0.23	158	0.82	-0.04
Horror	9.03	149	<0.003	0.74	0.43	159	0.67	0.03	3.71	152	<0.003	0.59
Mystery	1.35	149	0.18	0.11	-0.23	159	0.82	-0.02	3.26	158	<0.003	0.52
Romance	-33.52	149	<0.003	-2.74	2.43	159	0.02	0.19	-7.57	158	<0.003	-1.20
Science–Fiction	23.07	149	<0.003	1.88	0.78	159	0.44	0.06	9.08	158	<0.003	1.44
Thriller	6.77	149	<0.003	0.55	7.71	159	0.003	0.61	2.60	136	0.01	0.41
War	29.17	149	<0.003	2.38	-1.65	159	0.10	-0.13	7.17	158	<0.003	1.14
Western	24.22	149	<0.003	1.98	-9.89	159	<0.003	-0.78	4.35	152	<0.003	0.69

*Column (a) presents the results of comparing the mean ratings from Study 1 against an intermediate value of 5. Positive values of *t* and *g* indicate a ‘male’ genre, whereas negative values indicate a ‘female’ genre. Column (b) presents the results of comparing the mean preference ratings from Study 2 against an intermediate value of 5. Positive values of *t* and *g* indicate a popular genre, whereas negative values indicate an unpopular genre. Column (c) presents the results of comparing the preference ratings of male and female participants observed in Study 2. Positive values of *t* and *g* indicate a stronger male preference, whereas negative values indicate a stronger female preference. The significance criterion was set to 0.003 for each test using Bonferroni’s method for multiple comparisons. The degrees of freedom were corrected in column (c) if Levene’s test revealed a significant inequality of variances. Hedges’s *g* is given as an estimate of effect sizes (cf. Fritz et al., 2012).*

#### TV and Film Consumption

On average, the women in our sample watch TV for 1.9 h per day (*SD* = 1.0), view 2.0 films per week (*SD* = 1.7), and go to the cinema once in a month (*SD* = 0.8). On average, the men in our sample watch TV for 2.0 h per day (*SD* = 1.2), view about 3.0 films per week (*SD* = 3.5), and also go to the cinema once in a month (*SD* = 1.1). The results for men and women were not significantly different, all *t*s(149) < 1.10, all *p*s > 0.25, all *g*s < 0.40.

### Discussion

In Study 1, we investigated the stereotypes of young adults about the preferences of men and women with regard to 17 film genres. For each genre, our participants indicated the extent to which this genre is preferred by women or men. The results showed that five of the 17 genres were stereotyped as ‘female’ genres (animation, comedy, drama, ‘heimat’, romance), whereas ten genres were stereotyped as ‘male’ genres (action, adventure, erotic, fantasy, history, horror, science fiction, thriller, war, and Western). Two genres were considered as equally popular among men and women (crime, mystery).

Participants considered dramatic and romantic movies as ‘female’ genres, and they considered action-packed and violent movies (e.g., action, adventure, horror, thriller, war, and Western) as ‘male’ genres. Thus, our data reflect the most popular stereotypes about movie preferences of men and women and are, furthermore, in line with the results of previous research on gender differences in movie preferences (Sparks, 1991; Oliver et al., 1998; Harris et al., 2000; Krcmar and Kean, 2005).

In addition, we observed some less obvious or less familiar stereotypes. Animation and ‘heimat’ movies were stereotyped as female genres, whereas erotic, fantasy, history, and science fiction movies were stereotyped as male genres. The latter findings are possibly not so surprising because many fantasy, history, and science fiction movies contain action, competition, and violence—the features that are seen as more attractive to male viewers than to female viewers (e.g., Oliver, 2000). Interestingly, only two genres (crime, mystery) were viewed as equally liked by men and women. Finally, the empirical distinction between crime film (neutral) and thriller (male) is notable because these genres are sometimes collapsed into a single category (e.g., Fischoff et al., 1998).

When comparing the stereotypes of men and women about the movie preferences of men and women, we observed a striking degree of agreement. This agreement is reflected in the lack of a significant two-way interaction in the ANOVA. Moreover, across the 17 genres addressed in our study, the average judgments of men and women were correlated by *r* = 0.99.

## Study 2: Actual Movie-Genre Preferences of Men and Women

In Study 2, we investigated the actual preferences of young adults with regard to the 17 movie genres, which had already been addressed in Study 1. Our interest was threefold. We were, firstly, interested in the relative popularity of different film genres. Secondly, we wanted to further explore gender differences in the popularity of movie genres. And, thirdly, we were interested in comparing gender differences in actual film preferences to the stereotypes about gender differences in film preferences as observed in Study 1.

To our knowledge, Fischoff et al. (1998)^6^ reported the only study that explored a relatively large set (i.e., 14) of movie genres. They asked 560 participants for their favorite movie titles and then sorted the responses into genres. Results showed that film drama (31% of the titles), action movies (17%), romantic movies (13%), and comedy movies (11%) were the most popular genres across gender and age. In contrast, biblical films (0.5%) and documentaries (0.1%) turned out as the two least popular genres. Unfortunately, the procedure used by Fischoff et al. (1998) does not allow for comparisons between genres because it ignores differences in base rates (i.e., different number of films per genre). To illustrate that point, imagine that the film industry releases 100 dramatic movies, 50 romantic movies, and only 1 documentary each year. As a result, even a person who likes documentaries much more than dramas will probably include more dramas than documentaries in his/her list of favorite films simply because more dramas are available (for recall) than documentaries (e.g., Tversky and Kahneman, 1974). Thus, comparing the frequencies of titles from different genres leads to wrong conclusions about relative genre preferences. If we want to compare the popularity of different movie genres in a more valid way, we must ask participants to directly indicate their sympathy for each genre, and this is exactly what we did in our Study 2.

### Methods

#### Participants and Interviewer

One hundred and sixty volunteers participated in Study 2. The sample consisted of 80 women (mean age = 23.6 years; *SD* = 3.7) and 80 men (mean age = 23.5 years, *SD* = 3.2). The large majority of participants were again students (undergraduates) at a public university in Germany. The interviewers were six female students.

#### Materials

We developed a new questionnaire with 24 items^7^. Items 1–4 asked for demographic characteristics of our participants (gender, age, major study course, minor study course). Items 5–21 asked the participant, for each of 17 movie genres, to indicate on an 11-point rating scale how much they liked a particular genre. In particular, each item asked “How much do you like (horror) movies on a scale from 0 (not at all) to 10 (extremely)? Please tick.” The items 5–21 probed for the same genres as in Study 1. The items 22–24 asked our participants for some quantitative aspects of TV and film consumption as in Study 1.

We constructed two version of our questionnaire in order to minimize the effects of item order. For version A, items 5–21 were jumbled to produce a random order of genres. For version B, the random order from version A was reversed. Then we made sure that equal numbers of men and women filled out each version of the questionnaire.

#### Procedure

The procedure for Study 2 was the same as for Study 1.

### Results

#### Film-Genre Preferences of Men and Women

**FIGURE 2:**
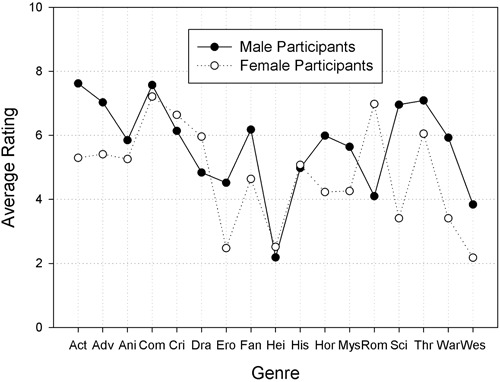
**Results of Study 2: Average ratings from 80 males and 80 females participants who indicated their preference for 17 movie genres on a rating scale with 11 ticks ranging from 0 (not at all) to 10 (extremely).** Short-hands on the *x*-axis are the same as in **Figure 1**

A preliminary analysis revealed that item order (i.e., the two variants of the questionnaire) had no effects on the results (all *F*s < 1, all *p*s > 0.25). Hence, we excluded this variable from further analyses, and conducted a two-factorial ANOVA on the mean preference ratings, which involved the between-subject factor *Gender of Participant* and the within-subject factor *Genre*. There were significant main effects for *Gender of Participant*, *F*(1,158) = 45.04, *MSE* = 12.45, *p* < 0.001, $\eta_{P}^{2}$ = 0.22, and *Genre*, *F*(12,1878) = 46.03, *MSE* = 7.96, *p* < 0.001, $\eta_{P}^{2}$ = 0.23. Moreover, there was also a significant two-way interaction, *F*(12,1878) = 16.36, *MSE* = 7.96, *p* < 0.001, $\eta_{P}^{2}$ = 0.09. **Figure 2** shows the corresponding means.

The main effect for *Gender of Participant* resulted from the fact that male participants (*M* = 5.67, *SD* = 2.42) liked most of the 17 genres more than the female participants (*M* = 4.77, *SD* = 2.59). In order to understand the main effect of *Genre*, we compared the mean rating for each genre to the neutral value of 5. Because multiple tests increase the risk of making a *type-1* error, we again used Bonferroni’s method to reduce the significance criterion α from 0.05 to 0.003. A significant positive deviation from the neutral value of 5 reflects a popular genre (cf. **Figure 2**). Five genres were popular: action, adventure, comedy, crime, and thriller. For nine genres, popularity did not differ from 5, and therefore these genres had average popularity: animation, fantasy, drama, history, horror, romance, mystery, science fiction, and war. A significant negative deviation from the neutral value of 5 signifies an unpopular genre (cf. **Figure 2**). Three genres were unpopular: erotic, ‘heimat’ movies, and Western. The results of the corresponding *t*-tests are presented in **Table 1** (column b).

To uncover the source of the significant two-way interaction of *Gender of Participant* and *Genre*, we compared the average male rating to the average female rating for each genre. For these comparisons, we again reduced the significance level to 0.003 by applying Bonferroni’s method. Drama and romance were more strongly preferred by women than by men. Nine genres were more strongly preferred by men than by women: action, adventure, erotic, fantasy, horror, mystery, science fiction, war, and Western. Finally, six genres were equally popular among men and women: animation, comedy, crime, ‘heimat’, history, and thriller. The results of the corresponding *t*-tests are presented in **Table 1** (column c).

#### TV and Film Consumption

On average, the women in our sample watch TV for 2.0 h per day (*SD* = 1.2), view 2.2 films per week (*SD* = 1.9), and visit the cinema 1.3 times in a month (*SD* = 1.2). On average, the men in our sample watch TV for 1.9 hours per day (*SD* = 1.4), view about 2.5 films per week (*SD* = 2.0), and visit the cinema once in a month (*SD* = 0.9). The results for men and women were not significantly different, all *t*s(158) < 1.55, all *p*s > 0.10, all *g*s < 0.25.

#### Comparison between Studies 1 and 2

We compared presumed and actual movie preferences of men and women in qualitative and in quantitative terms. **Table 2** (fourth column) gives the concordance between stereotypical and actual gender differences in movie preferences for each genre in qualitative terms (“+” = match, “–“ = mismatch). The classification of genres as male, female or neutral from Study 1 agreed with the classification of genres from Study 2 for 11 of 17 genres (i.e., for 65% of genres). This agreement was larger for ‘male’ genres (8 from 10 genres) than for ‘female’ genres (2 out of 5 genres). The majority (i.e., 5 out of 6 genres) of mismatches were overestimations, that is, participants presumed a gender gap in movie preferences that was actually not present.

**Table 2:** A comparison of gender stereotypes about the popularity of 17 movie genres and actual film preferences of men and women.

Genre	Study 1:	Study 2:	Study 1 vs.	Study 2:
	Stereotype	Preference	2 compared	Rank
Action	Men	Men	+	5
Adventure	Men	Men	+	4
Animation	Women	Neutral	–	6
Comedy	Women	Neutral	–	1
Crime	Neutral	Neutral	+	3
Drama	Women	Women	+	8
Erotic	Men	Men	+	15
Fantasy	Men	Men	+	9
‘Heimat’	Women	Neutral	–	17
History	Men	Neutral	–	12
Horror	Men	Men	+	11
Mystery	Neutral	Men	–	13
Romance	Women	Women	+	7
Science-Fiction	Men	Men	+	10
Thriller	Men	Neutral	–	2
War	Men	Men	+	14
Western	Men	Men	+	16

*The second column shows the gender that participants thought of having the stronger preference for a genre (Study 1). The third column shows the gender that actually had the stronger preference for a genre (Study 2). The fourth column shows whether the presumed gender difference and the actual gender difference matched (indicated by +) or mismatched (indicated by –). Finally, the right-most column contains the ranks of movie genres regarding their popularity, averaged across gender.*

**FIGURE 3:**
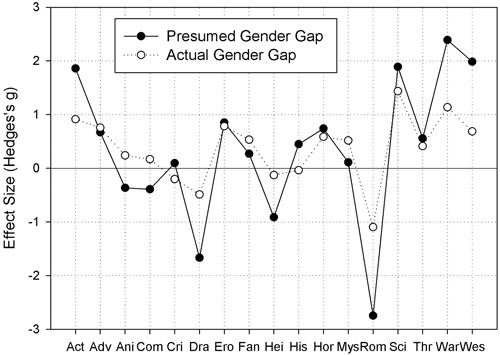
**Black points show the effect sizes (Hedges’s *g*) of the presumed gender gap in movie preferences as observed in Study 1.** Negative values indicate a stronger presumed female preference for a genre, whereas positive values indicate a stronger presumed male preference. White points show the effect sizes (Hedges’s *g*) of the actual gender gap in movie preferences as observed in Study 2. Negative values indicate a stronger female preference for a genre, whereas positive values indicate a stronger male preference. Short-hands on the x-axis are the same as in **Figure 1**

**Figure 3** presents a quantitative picture of the concordance between stereotypical and actual gender differences in movie preferences. We compared the effect sizes (i.e., Hedges’s *g*; cf. Ferguson, 2009; Fritz et al., 2012) of presumed gender differences in movie preferences to the effect sizes of actual gender differences in movie preferences. The black points in **Figure 3** show the effect sizes of the presumed gender gap in movie preferences as observed in Study 1 (the actual numbers are given in column a of **Table 1**). Each point represents the difference between the preference rating for a genre and the neutral value of 5. Hence, negative values indicate a presumed female preference for a genre, whereas positive values indicate a presumed male preference. The white points in **Figure 3** show the effect sizes of the actual gender gap in movie preferences as observed in Study 2 (the actual numbers are given in column c of **Table 1**). Each point represents the difference between the preference ratings of men and women for a genre. Hence, negative values indicate an actual female preference for a genre, whereas positive values indicate an actual male preference. The patterns appear quite consistent: In fact, the correlation between the presumed gender differences and actual gender differences (across genres) is very high: *r* = 0.92 (for effect sizes). However, there were numerical differences between the (effect size of) presumed gender differences and the (effect size of) actual gender differences in movie preferences.

We classified a difference as an overestimation when the presumed gender gap exceeded the actual gap by more than 0.4 in effect size, and we classified a difference as an underestimation when the actual gender gap exceeded the presumed gap by more than 0.4 in effect size^8^. If the difference between presumed and actual gender difference was smaller than 0.4 in effect size, we counted the case as a match. According to this scheme, there were ten overestimations (sorted alphabetically: action, animation, comedy, drama, ‘heimat’, history, romances, science fiction, war, Western), one underestimation (mystery), and six matches (adventure, crime, erotic, fantasy, horror, thriller; cf. **Figure 3**).

### Discussion

Study 2 investigated the popularity of 17 movie genres among young adults. There were three general findings. Across both genders, we observed marked differences in the relative popularity of the 17 genres. The popularity of five genres was significantly above the intermediate value of 5: comedy, thriller, crime, adventure, and action (sorted by rank order; cf. **Table 2**, rightmost column). When compared to the results of Fischoff et al. (1998), we notice that crime and thriller were among the less popular genres in their study. The difference between the results of their and our study may reflect cultural differences between Germany and the U.S., or may reflect a methodological problem in their study, namely, the possible impact of different base rates (i.e., different number of films per genre; see above) on the frequency of naming movie titles from different genres (Fischoff et al., 1998). Also, as the study by Fischoff et al. (1998) was conducted almost 20 years ago, it might be that preferences for movie genres have changed over time. In our study, nine genres were of intermediate popularity: animation, romance, drama, fantasy, science fiction, horror, history, mystery, and war (sorted by rank order). And there were three unpopular genres (i.e., their average popularity was significantly below the intermediate value of 5): erotic, Western, and ‘heimat’ films (also sorted by rank order).

Study 2 also revealed gender differences in the popularity of most movie genres. Half of the genres (i.e., 9 from 17) were preferred by men. The following list is ordered by the effect size of the gender gap from large to small (the effect sizes are given in **Table 1** and depicted in **Figure 3**): science fiction, war, action, erotic, adventure, Western, horror, fantasy, and mystery. Six genres were equally popular among men and women (sorted by absolute popularity from high to low): thriller, animation, comedy, history, ‘heimat’ films, and crime. Only two movie genres were preferred by women: romance and drama.

Another goal of our study was to compare stereotypes about film preferences of men and women, as observed in Study 1, to actual film preferences of men and women, as observed in Study 2. Our results revealed that for the majority of movie genres under investigation the participants (in Study 1) correctly predicted the existence or direction of a gender gap in movie preferences (as observed in Study 2), but overestimated the size of the gender gap. We discuss this finding in some more detail in the Section “General Discussion”.

## General Discussion

In two independent studies, we investigated gender stereotypes about film preferences of men and women (Study 1) and actual film preferences of men and women (Study 2) with regard to 17 movie genres. Study 1 revealed gender stereotypes for the large majority (i.e., 15 out of 17) of genres under investigation (cf. **Table 2**, second column). The findings reflect the most popular clichés about gender differences in movie preferences. Interestingly, male and female participants reported very similar gender stereotypes on film preferences. In fact, across the 17 genres, the average ratings from men and women correlated with *r* = 0.99, an outstanding correlation. Study 2 assessed the actual movie preferences in a new sample of men and women. As a first result, the study revealed gender-independent differences in the popularity of 17 movie genres (cf. **Table 2**, rightmost column). In addition, we observed strong gender differences in the popularity of many genres. In fact, a gender effect was found for 11 out of 17 genres (cf. **Table 2**, central column). Only two genres were more strongly preferred by women, whereas nine genres were more strongly preferred by men. These findings replicate and extend previous findings on gender differences in movie preferences (e.g., Sparks, 1991; Oliver et al., 1998; Harris et al., 2000; Oliver, 2000; Krcmar and Kean, 2005; Greenwood and Lippmann, 2010). The fact that men and women reported similar quantitative patterns of media consumption (e.g., numbers of movies watched per week) rules out this variable as an explanation for differences in movie preferences.

The finding that a majority of genres is preferred by men may reflect a male dominance in the movie business. For example, Smith et al. (2013) analyzed gender inequality in 500 films released between 2007 and 2012 and found that, on average, 70% of the speaking characters on screen were male. Thus, assuming that observers enjoy films that allow them to identify with the major protagonist and that observers prefer to identify with same-sex protagonists (e.g., Hoffner and Cantor, 1991; Hoffner, 1996), the preponderance of male protagonists in films may explain why men prefer more genres than women in our study.

### The Accuracy of Gender Stereotypes in Movie Preferences

This is the first empirical study that investigated the accuracy of gender stereotypes about movie preferences. Therefore, we assessed stereotypes about movie preferences of men and women in Study 1 and compared them to the actual movie preferences of men and women as observed in Study 2. This comparison revealed three main findings. First, the participants correctly predicted the *direction* of gender differences in movie preferences for the majority of genres (i.e., for 65% of the genres). Second, the participants overestimated the *size* of gender differences in movie preferences for the majority of genres: The predicted gender gap exceeded the actual gap by more than 0.4 effect sizes for 10 out of 17 (59%) movie genres. Third, male and female participants made very similar predictions about the movie preferences of men and women (cf. **Figure 1**) and, hence, the stereotypes of men and women were similarly accurate. We may conclude then that gender stereotypes regarding movie preferences were accurate in direction, but inaccurate in size.

Indeed, the finding that our participants correctly predicted the direction of gender differences in preferences for most movie genres under investigation also fits the majority of findings from previous studies on the accuracy of gender stereotypes. In fact, from their literature review, Jussim et al. (2009) conclude that the majority of judgments on attitudes and abilities of men and women can be considered as accurate. However, our finding that our participants overestimated the size of gender differences in movie preferences for the majority of genres seems less consistent with the literature. In fact, if studies observed overestimations of gender differences at all, then they occurred for a minority of attributes, and were often counterbalanced by underestimations (e.g., Swim, 1994; Beyer, 1999; Halpern et al., 2011). So, the question arises: Why did participants generally overestimate gender differences in movie preferences in our study? A possible reason may be that movie advertisements (and movies) often target a particular gender, and therefore suggest stronger differences in movie preferences than actually exist. Another possible reason for the discrepancy between the underestimation of actual gender differences in (cognitive) abilities (e.g., Halpern et al., 2011) and the overestimation of gender differences in movie preferences in our study may be that social desirability (or political correctness) has a larger impact on how people think or talk about gender differences in abilities than on how people think or talk about gender differences in (movie) preferences.

### Practical Implications

Our empirical results showed that several beliefs about gender differences in movie preferences are inaccurate in that they overestimate actual gender differences. These results could be used for reducing (or eliminating) gender stereotypes and, hence, for improving the relationship between groups (e.g., Beyer, 1999). For example, both men and women erroneously believed that women have a stronger preference for ‘heimat’ movies than men, although both groups actually disliked this genre to a similar degree. If confronted with this stereotype, women may respond negatively because the assumed preference for ‘heimat’ movies may be associated with a traditional gender role or rather old-fashioned attitude. To give another example, the participants strongly overestimated existing gender gaps in preferences for the most stereotypical genres (i.e., action, science fiction, and war movies as ‘male’ genres vs. drama and romance as ‘female’ genres). In other words, women like typical ‘female’ genres to a lesser degree than presumed and men like typical ‘male’ genres to a lesser degree than presumed. As a result, people may often overestimate the preference of a particular individual for a gender-congruent genre, which may produce some discomfort in that individual. That discomfort could be avoided if people were aware that gender differences in movie preferences, if present at all, are smaller than presumed.

Our results could also be informative for the movie industry. Modern movies and the advertisement for these movies are often conveying gender stereotypes, and depicting traditional gender roles (e.g., Greenwood and Lippmann, 2010). First, our data are informative with regard to the popularity of movie genres across viewer gender. In particular, we found that, at least for our German student population, comedy, thriller, and crime movies are the most popular genres, whereas erotic, Western, and ‘heimat’ movies are the least popular genres. Second, our data are informative with regard to gender differences in movie preferences. In particular, the largest gender gaps (with effect sizes >1) occurred for science fiction and war movies, which were strongly preferred by men, and romantic movies, which were strongly preferred by women. Here the question arises whether gender differences in actual preferences are so large because the contents of genre-typical movies are the most stereotypical, or because these genres most strongly address the particular interests of each gender. Empirical results on actual gender differences in interests provide some support for the latter hypothesis (e.g., Lippa, 2010). In particular, research shows that women are, on average, more strongly interested in people (and relationships) than men (see Su et al., 2009), matching their preference for romantic movies. In contrast, men are, on average, more strongly interested in things (i.e., technical equipment) than women (see Su et al., 2009), probably matching their preference for science fiction movies. The implication might be that it could be very difficult to attract (more) women to science fiction and war movies, or to attract (more) men to love and romantic films. Third, if the industry would like to produce movies that similarly appeal to both genders, they should produce comedy, crime, or even thriller movies. These were not only the most popular genres, but they were equally preferred by men and women—in contrast to the stereotype of ‘female’ comedy and ‘male’ thriller.

### Limitations of the Study and Directions for Further Research

Our study is limited with regard to (a) the set of movie genres included in our study and (b) the population under investigation. Although we investigated a larger set of movie genres than previous studies, we still excluded some interesting movie genres, such as documentaries, sports movies or musicals. Moreover, we also excluded hybrid genres (e.g., romantic comedy) and sub-genres (e.g., dark comedy) from our investigation. Thus, it is possible that our participants in fact thought of different sub-genres when faced with a particular genre name, and averaging across such heterogeneous categories should have increased the between-subjects variance in our data. It might be interesting to do follow-up investigations on the accuracy of gender stereotypes about preferences for different sub-types of a movie genre.

Also, future research could use different questionnaire items. In our first study, for instance, participants where asked how much they thought a film of a certain genre was preferred by men or by women. Instead, one could ask participants for assumed gender-specific preference for certain film features (e.g., whether the film ends on a happy end). Thus, one could avoid the use of genre, because this term might serve as a strong label and thus a category that evokes gender stereotypes.

Our two studies assessed movie preferences in two samples of young students, aged between 18 and 35 years, enrolled in different majors at a German University. There is evidence that movie preferences differ between age groups (e.g., Fischoff et al., 1998), and between societies or cultures (e.g., Germany vs. India; e.g., Khanna, 2003). In addition, the concept of a movie genre cannot be easily transferred across cultures. For example, whereas a single genre typically dominates American and European movie productions, typical movies from India combine elements from different genres such as action, comedy, dance, music, and romance (Uhl and Kumar, 2004). A main reason for this difference is that Indian movies are intended to entertain the whole family, whereas this is not the case for most American and European movies. Hence, there is reason to believe that, similar to many other findings of psychological research, our findings are restricted to western, educated, industrialized, rich, and democratic (WEIRD) societies (Henrich et al., 2010). Finally, because the majority of University students in Germany belong to the middle and upper class of the Germany society, our results are restricted to these groups as well.

Finally, future studies might investigate how participant’s gender role identity (as measured, for example, with the sex-role inventory; Bem, 1974) might influence movie preferences. One might expect a positive correlation between masculinity and the preferences for male movie genres, and also between femininity and the preferences for female movie genres. Further, one might expect that androgynous persons exhibit relatively weak gender-related preferences for movie genres.

With regard to stereotypes of gender difference in movie preferences we would expect interesting insights from considering attitudes towards men and women too (as measured, for example, with the Ambivalent Sexism Inventory; Glick and Fiske, 1996). One might expect that negative attitudes toward women (as reflected by high scores in hostile sexism) could lead to a devaluation of female genres, and thus increasing the gender gap for the female genres. Further studies are needed to investigate these predictions directly.

## Conclusion

This is the first empirical investigation of gender stereotypes about movie preferences, and a first empirical test of the accuracy of gender stereotypes of movie preferences. We observed that people hold gender stereotypes about the movie preferences of men and women for a wide range of movie genres. Interestingly, men and women hold very similar gender stereotypes about the movie preferences of men and women. When compared to actual movie preferences of man and women, gender stereotypes about movie preferences were accurate in direction, but inaccurate in size. In particular, gender stereotypes about movie preferences overestimated actual gender differences in movie preferences for the majority of genres that we investigated. The latter finding is particularly interesting because previous research on the accuracy of gender stereotypes found that people generally underestimate actual gender differences in (cognitive) abilities (e.g., Halpern et al., 2011). Finally, the results of our study have some practical implications. For example, the fact that stereotypes overestimated actual gender differences in movie preferences could be used for reducing the stereotypes. In addition, the results of our study may be useful for the movie industry in identifying movie genres that are equally attractive to men and women.

## Author Contributions

SS and PW planned the study, prepared the materials, and supervised data collection. PW analyzed the data. BL, SS, and PW wrote the manuscript.

## Conflict of Interest Statement

The authors declare that the research was conducted in the absence of any commercial or financial relationships that could be construed as a potential conflict of interest.

## Footnotes

1 The German term ‘heimat’ can be translated into ‘home’ or ‘home country’. It refers to the area or country where you are born, live or feel “at home”. There are two different variants of the German ‘heimat’ film. The classic type (~1950–1970) typically described rural life in an idealized or romanticized way. In contrast, the modern type of ‘heimat’ film (after 1970) typically described rural life in a realistic manner.

2 Beyer (1999) defined a major as ‘masculine’ when participants believed that the majority of students in that major were male (i.e., computer science, chemistry, business, history, mathematics, political science, and biology).

3 A list of typical movie genres can be found in the Internet Movie Database (http://www.imdb.com/genre).

4 The original questionnaires are available from the authors upon request.

5 For both studies reported here, the results of all *F* tests were corrected according to Greenhouse and Geisser (1959), if Mauchly’s test of sphericity was significant. The Greenhouse–Geisser correction reduces the degrees of freedom, making the *F* test more conservative. In addition, we report $\eta_{P}^{2}$ as a measure of effect size in *F* tests, and Hedges’s *g* as a measure of effect size in *t*-tests (cf. Fritz et al., 2012).

6 Please note that this study was published in the internet, and not in a peer-reviewed journal.

7 The original questionnaires are available from the authors upon request.

8 In choosing .40 as a cut-off value, we follow Ferguson (2009, p. 533) who recommended that *g* = 0.41 is the minimum effect size with a “practical” significance for social science research.
